# Gender differences in the associations of circadian function with cognition among older persons with sleep disturbances

**DOI:** 10.1002/alz.089617

**Published:** 2025-01-09

**Authors:** Yulu Pan, Gawon Cho, Margaret Doyle, Lakshmi Polisetty, Chase Burzynski, Lynne Iannone, Melissa Knauert, Henry Yaggi, Thomas M. Gill, Brienne Miner

**Affiliations:** ^1^ Yale School of Medicine, New Haven, CT USA; ^2^ Bridgeport Hospital, Bridgeport, CT USA; ^3^ Yale University, New Haven, CT USA

## Abstract

**Background:**

Circadian function, characterized by circadian strength, timing, and fragmentation, has been shown to correlate with cognitive function; however, little is known about in these associations, particularly among older adults who may have more variability in their sleep‐wake schedules.

**Method:**

Among 60 community‐living adults aged 60‐90 years with self‐reported sleep complaints ≥once/ week, we investigated gender differences in the associations between circadian and cognitive function. Participants completed seven days of actigraphy and one night of polysomnography. Circadian function variables were calculated using activity counts from actigraphy and included: circadian strength (amplitude, interdaily stability [IS; degree of consistency of activity patterns from day‐to‐day], mesor); timing (activity peak time, sleep midpoint [midpoint between sleep onset and offset]); and fragmentation (intradaily variability [IV: fragmentation of the rhythm relative to its 24‐h amplitude]). Cognitive function was assessed by the Montreal Cognitive Assessment (MoCA; higher scores indicate better cognitive function). Pearson correlation coefficients examined the associations between circadian variables and cognitive function in men, women, and all participants, adjusting for age, race, and total sleep time (from polysomnography).

**Result:**

The mean age was 74 (±6.4) years, with 65% women and 33.3% minority race. Among women, higher IS (r=0.62, p<0.001) and lower IV (r=‐0.44, p=0.006) significantly correlated with higher MoCA score. Among men, earlier sleep midpoint (r=‐0.53, p=0.02) and earlier activity peak time (r=‐0.53, p=0.02) significantly correlated with higher MoCA score. The amplitude‐MoCA score association was non‐significant in women (r=0.31, p=0.07) and men (r=0.30, p=0.21) but significant in all participants (r=0.32, p=0.01). No significant associations were found between mesor and MoCA score in any group.

**Conclusion:**

Among community‐dwelling older adults with sleep disturbances, circadian strength and fragmentation [IS, IV] correlated with cognitive function only in women, while circadian timing [activity peak time, sleep midpoint] correlated with cognitive function only in men. These differences may be related to sex differences in other sleep‐related factors, such as time in slow‐wave‐sleep and melatonin amplitude, which are higher in women than men. Further investigation is needed to understand biological differences that may explain the differential association of circadian variables with cognition in older men and women.

